# A Non Linear Scoring Approach for Evaluating Balance: Classification of Elderly as Fallers and Non-Fallers

**DOI:** 10.1371/journal.pone.0167456

**Published:** 2016-12-09

**Authors:** Julien Audiffren, Ioannis Bargiotas, Nicolas Vayatis, Pierre-Paul Vidal, Damien Ricard

**Affiliations:** 1 CMLA, ENS Cachan, CNRS, Université Paris-Saclay, Cachan, France; 2 COGNAG-G UMR 8257 CNRS, SSA, Université Paris Descartes, Paris, France; 3 Service de neurologie, HIA Percy, Service de Santé des Armées, Clamart, France; 4 Ecole du Val-de-Grâce, Service de Santé des Armées, Paris, France; Tokai University, JAPAN

## Abstract

Almost one third of population 65 years-old and older faces at least one fall per year. An accurate evaluation of the risk of fall through simple and easy-to-use measurements is an important issue in current clinic. A common way to evaluate balance in posturography is through the recording of the centre-of-pressure (CoP) displacement (statokinesigram) with force platforms. A variety of indices have been proposed to differentiate fallers from non fallers. However, no agreement has been reached whether these analyses alone can explain sufficiently the complex synergies of postural control. In this work, we study the statokinesigrams of 84 elderly subjects (80.3+− 6.4 years old), which had no impairment related to balance control. Each subject was recorded 25 seconds with eyes open and 25 seconds with eyes closed and information pertaining to the presence of problems of balance, such as fall, in the last six months, was collected. Five descriptors of the statokinesigrams were computed for each record, and a Ranking Forest algorithm was used to combine those features in order to evaluate each subject’s balance with a score. A classical train-test split approach was used to evaluate the performance of the method through ROC analysis. ROC analysis showed that the performance of each descriptor separately was close to a random classifier (AUC between 0.49 and 0.54). On the other hand, the score obtained by our method reached an AUC of 0.75 on the test set, consistent over multiple train-test split. This non linear multi-dimensional approach seems appropriate in evaluating complex postural control.

## Introduction

Human ability to stand and walk is mainly the result of postural control. Postural control is achieved by a complex synergy of namely the visual, proprioceptive, and vestibular systems, as well as the central nervous system [1]. The combination of skeletal, visual, balance and gait impairments throughout age may lead to a correspondingly significant loss of postural control, which increases the risk of falling [2]. It has been shown that falls are considered as one of the major causes of injury in elderly, leading to further restriction in mobility, loss of autonomy in daily activities (dressing, bathing etc) or even death [3, 4]. The fact that almost one third of population 65 years old and older faces at least one fall per year [4] makes the early identification, prediction and prevention of falls crucial.

In current clinic, postural control in community-dwelling elderly population is evaluated using balance assessment scales. These scales are easy to use, fitting well with the requirements of daily clinic and they have been proved to be extremely useful to clinicians in evaluating balance control [5–7]. However, their final effectiveness in predicting falls in community-dwelling older adults has been recently questioned and a careful use of their results has been advised [7–9]. Additionally, in many balance assessment scales, the measurement of individuals’ progress is very difficult [10].

To overcome those difficulties, clinical researchers use force plate platforms to evaluate postural control during quite stance. These platforms measure the displacement of centre of pressure (CoP) in time [11] and quantify the balance control. This measurement is usually called statokinesigram, stabilogram or sway. Statokinesigrams have been widely used in balance disorder studies not only in elderly populations [12] but also in widespread diseases such as Parkinson’s disease [13]. One of the well-known procedures that are followed in elderly subjects includes quite stance (feet apart) for 10-60 s with 1) eyes open (hands by the side) and 2) eyes closed (hands by the side) [3, 14]. The principle of this procedure is that individuals with postural instability become significantly visually reliant and the closed-eyes acquisition may amplify the differences between high and low risk individuals [15].

With the wider availability of CoP platforms, there is an increasing interest in extracting additional information from the statokinesigrams. Previous works have proposed a variety of empirical indices (or features) derived by the actual CoP displacements (such as the area covered by the CoP’s trajectory or sway area, mean distance of points from centre of CoP trajectory, velocity and acceleration of trajectory), showing that in some cases sway area, mean velocity or mean acceleration are affected by instability [3, 16]. Other approaches used indices derived from CoP-trajectory transformations such as Fourier [17, 18], Wavelet [19] or Sway density [17, 20]. However, as it has been reported in healthy and non healthy populations [21, 22], no agreement has been reached whether these approaches alone can provide enough information to characterize the complex synergies of postural control.

Our approach is inspired by the latter absence of consensus. Indeed, standard measurements such as sway combined with simple, linear statistical tools can hardly describe highly complex phenomena such as postural control. In our case, we hypothesized that more complex non linear models could be more fruitful in order to exploit the interrelations of many indices, which might characterize fallers/non-fallers statokinesigrams.

The objective of this work is to build such model using a multi dimensional description of the statokinesigram combined with a Ranking Forest [23], a machine learning approach, which will be able to significantly distinguish fallers/non-fallers in community-dwelling elderly population. In order to achieve this objective, our work followed two main steps:

- the description of the individuals’ statokinesigrams (open & closed eyes) through a multi-dimensional description (5 features) and
- the analysis of the performances of the Ranking Forest scoring algorithm [23] to non-linearly combine these features.

Our goal is to provide an accurate, consistent and easily interpretable score, as a potential addition to the well-established assessment tests. There are not many works that used advanced data mining in posturographic data and to our knowledge, the Ranking Forest algorithm has not been applied yet to such data.

## Materials and Methods

### Participants

Participants were recruited in different sites, the Neurology department of the Val-de-Grace hospital (Paris, France), the emergency department of the Bégin hospital (Paris,France), and the consultation office of a practitioner (Paris, France). Inclusion criteria were (1) age > 65 years; (2) addressed in consultation in general medicine or neurology; (3) could stand on the platform, (4) did not suffer from balance related impairment (5) gave informed consent. Particularly,only healthy individuals—i.e. asymptomatic individuals after clinical examination—were considered in this study. Individuals which were significantly hypertensive (mean Systolic Blood Pressure (SBP) ≥ 140 mmHg or mean Diastolic Blood Pressure (DBP) ≥ 90 mmHg), hypotensive (SBP ≤ 90 mmHg or DBP ≤ 60 mmHg), had particular impairements or used medication which could alter significantly their balance (such as vasoactive, phychotrope drugs) were excluded.

### Standard protocol approvals, registrations, and patient consents

The clinical Research Ethics Committee approved the clinical study, registered at ANSM (ID RCB 2014-A00222-45). Written informed consent was obtained from all participants.

### Balance measurements

The statokinesigrams were recorded using a custom application, in Android, specially developed for the study. Balance measurements were acquired using a Wii Balance Board (Nintendo, Kyoto, Japan) which has been found a suitable tool for the clinical setting with acceptable accuracy [24, 25]. We proceeded as follows: the individual was asked to remove his/her shoes, and to step on the platform. The feet were placed in the most comfortable position for the patient without exceeding the shoulder width. The individual was asked to stand in upright position (arms laying at the side) with open eyes. The displacement of the CoP was recorded for 25 seconds. Afterwards, the individual was asked to close his eyes, and after 10 seconds, the displacement of the CoP was recorded for 25 additional seconds.

### Fall assessment

A retrospective fall questionnaire was filled for each individual, asking how many times the participant recalled having fallen in the previous six months. Individuals were asked about the circumstances of the fall (if any)—particularly, if the patient was unbalanced by an external element– and how many of these falls resulted in injuries, hospitalizations or other trauma.

For this study, an individual was labeled as faller if he/she came to a lower level on the ground unintentionally [26], at least once, without being externally pushed or pulled, regardless of whether an injury was sustained [14].It is worth noting that in our population, fall-prone individual reported no more than two falls in the past six months.

### Statokinesigrams and Descriptors

**Fig 1:**
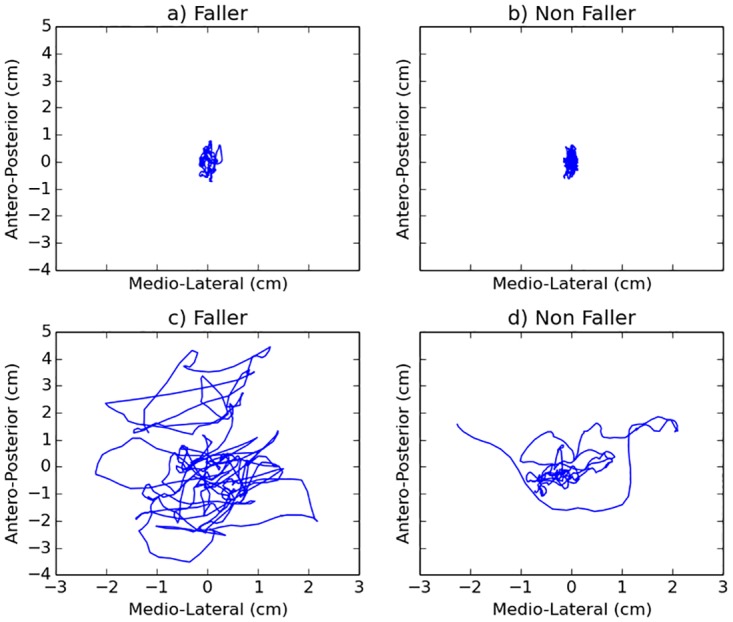
Examples of statokinesigrams from faller and non faller individuals, during the open eyes part of the experiment. Interestingly, fallers and non fallers do not always have visually distinctive statokinesigrams. A tight faller statokinesigram in a) seems pretty close to the non-faller’s statokinesigram in b). Similarly, a wider non-faller’s statokinesigram in c) seems close to a faller’s statokinesigram in d). In those examples, simple indices alone such as velocity of sway area or acceleration alone would probably fail to discriminate fallers from non fallers.

The statokinesigrams were obtained by sampling data from the Wii Balance Board at its inherent non-constant frequency during the record. The signal was then resampled at 25Hz using the SWARII algorithm (for more details on the non uniform sampling of the Wii Balance Board and the SWARII algorithm, we refer the reader to [27]). Fig 1 shows four examples of statokinesigrams obtained during the experiment. For each statokinesigram, five different transformations were computed as follows: 1) the position of the CoP along the medio-lateral axis, 2) the position of the CoP along the antero-posterior axis, 3) the distance between the position of the CoP and the center of the trajectory, or radius, 4) the instantaneous acceleration of the CoP and 5) the ballistic intervals, i.e. the distance between peaks of sway density, where the sway density is defined as the time necessary for the CoP to leave a small spatial circle centred around the current position [17, 20]. Then five indices where derived from those signals (the exact descriptors are defined in Table 1). They encode a five dimensional representation of the signal.

**Table 1:** Summary of the descriptors used.

Abbreviation	Description
**Time Domain**	
(*ρ*_*cl*_)_0.5_	Median of the radius of the statokinesigram, during the closed eyes record (*cm*).
(*a*_*cl*_)_0.1_	10-th percentile of the norm of the acceleration, during the close eyes record (*cm*.*s*^−2^).
$V(y_{cl})$	Variance of the values of the antero posterior coordinate of the signal, with close eyes. (*cm*^2^)
(*x*_*c*/*o*_)_0.1_	Ratio (close eyes over open eyes values) of the 10-th of the values of the medio lateral coordinate of the signal. (No unit)
**Sway Density**	
$V({\bar{i}}_{op})$	Variance of the ballistic intervals of the signal, during the open eyes record. (*s*^2^)

### Ranking Forest

In order to compute a score from the resulting five dimensional description of the statokinesigram, we used a classical machine learning approach on a train-test split: the dataset (the set of all the statokinesigrams collected with the faller/non faller label) is randomly split in a training set, containing 70% of the statokinesigrams, and a testing set containing the remaining 30%, while the proportion of faller and non faller were constrained to be the same for both set. We then used the Ranking Forest algorithm, a recent state-of-the-art scoring algorithm that is based on advanced probability and machine learning theory [23, 28]. The key idea behind this algorithm is the notion of *bagging*, i.e. in this case the construction and aggregation of many weak classifiers, here 50 decision trees, to obtain an efficient and robust predictor. Each decision tree is build on a randomly selected subset of the training dataset, while using only a randomly selected subset of the available features for the splitting of its nodes.

**Fig 2:**
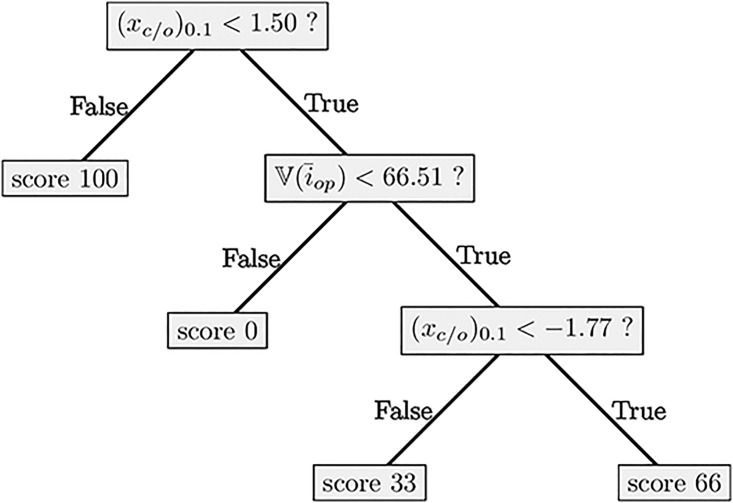
Example of a decision tree build by the Ranking Forest algorithm.

When evaluating a statokinesigram, each tree produces a score, and the scores of all the trees are averaged to produce the score of the Ranking Forest. The resulting score takes value between 0 and 100, 100 suggesting a high quality balance. An example of a decision tree constructed by this approach on our dataset can be found in Fig 2.

We propose an online version of the resulting scoring algorithm, which can be found at http://taureau.pppcmla.ens-cachan.fr/, and which can be applied to the existing dataset or to new statokinesigrams.

### Evaluation

As described in the previous subsection, in order to analyse the performance of the scoring approach, we use an usual train-test approach. The previously described Ranking Forest algorithm was trained on the training set, with 50 trees, with cross entropy as the objective function. Then, it was evaluated on the test set: for each statokinesigram in the test set, we compared the score computed by the algorithm with the declaration of falls of the patient, thus obtaining a receiver operating characteristic (ROC curve) of our scoring approach as well as the area under the ROC curve (AUC).

Additionally, for each descriptor, we reported the average value and the standard variation for both populations, fallers and non fallers, with the double tailed p-value of the non parametric Wilcoxon test (difference were significant when *p* < 0.05) and the AUC of each descriptor, which was computed by using a soft margin linear one dimensional Support Vector Machine.

## Results

Baseline data on age, sex, weight, height of the participants were collected and are presented in the Table 2.

**Table 2:** Demographic characteristics of the patients enrolled.

	Total Sample	Non Fallers	Fallers
Demographic	84	60	24
Male Female	40 44	27 33	13 11
Age (years) Weight (kg) Height (cm) BMI (kg.*m*^−2^)	80.3(±6.4) 70.0(±10.5) 167.1(±8.4) 24.90(±2.39)	79.8(±6.6) 70.1(±10.5) 167.2(±8.4) 24.95(±2.4)	81.3(±5.8) 68.5(±10.5) 167.0(±8.4) 24.78(±2.3)

Fallers are patient who declared at least one fall in the 6 previous months. No statistically significant difference was found between the two population regarding genre, age, weight, height and body mass index (BMI).

In our population, most of the features,except for (*x*_*c*/*o*_)_0.1_, did not show significant results through the conventional Wilcoxon test (*p* < 0.05), and their performances in ROC analysis was close to the performance of a random classifier (AUC between 0.49-0.54), questioning the features’ ability to successfully classify the two groups (Table 3).

**Table 3:** Mean and standard deviation for fallers and non-fallers, AUC, p-value for the Wilcoxon rank-sum test for each of the descriptors.

Descriptor	Mean(std) for fallers	Mean(std) for non fallers	AUC	p(Wilcoxon)
(*ρ*_*cl*_)_0.5_	0.69(±0.40)	0.53(±0.21)	0.49	0.37
$V({\bar{i}}_{op})$	71.48(±23.20)	95.21(±102.19)	0.53	0.62
(*a*_*cl*_)_0.1_	0.011(±0.009)	0.007(±0.004)	0.54	0.18
$V(y_{cl})$	0.77(±0.64)	0.42(±0.29)	0.52	0.09
(*x*_*c*/*o*_)_0.1_	−0.42(±2.77)	0.78(±1.87)	0.53	0.008
RKF	-	-	0.75	-

In Table 3 we see that indices alone cannot classify fallers non fallers. This might result from the fact that, as Fig 1 illustrates, in our population, some fallers and non fallers have very similar statokinesigrams. For instance, some fall-prone individuals have a characteristically larger statokinesigrams, and a high variance of the antero-posterior coordinates, while others may have a narrow CoP trajectory—see Fig 1a) and 1c). In Table 3, we observed that those inherently contradictory properties challenge the classification accuracy of the linear approaches, as the methods tend to wrongly label one or more categories of fallers.

Additionally, although the use of the feature (*x*_*c*/*o*_)_0.1_ alone produces an AUC close to random, it showed a significant *p*-value in Wilcoxon test (see Table 3).

**Fig 3:**
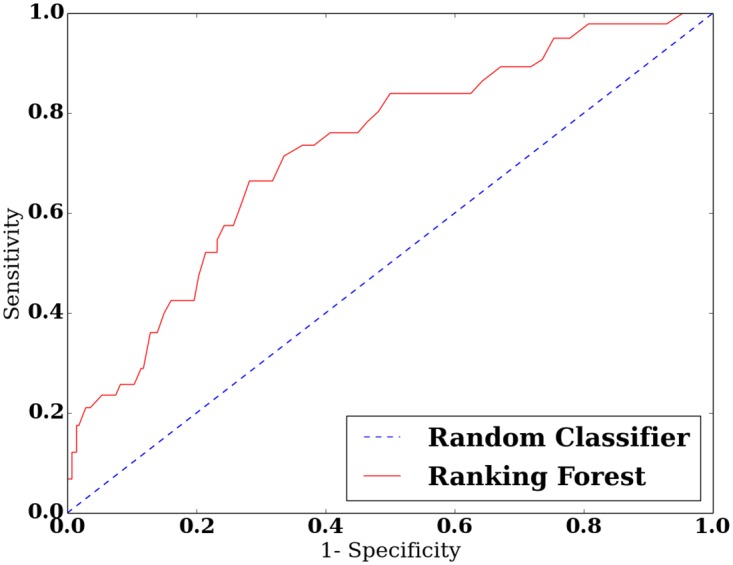
ROC curve of the score obtained with the Ranking Forest approach.

Conversely, the Ranking Forest approach, which combines all the descriptors in a complex, non linear manner obtains a significant AUC of 0.75. The ROC curve obtained by this methods is presented in Fig 3.

Finally, Table 4 shows the average relative feature importance derived from the Ranking Forest algorithm. It is worth noting that all the features were considered important by the algorithm, which might emphasize the necessity of using multiple different features when analyzing statokinesigrams.

**Table 4:** Mean and standard deviation of the feature importance (Feat. Imp.) as derived from the Ranking Forest algorithm.

	(*ρ*_*cl*_)_0.5_	$V({\bar{i}}_{op})$	(*a*_*cl*_)_0.1_	$V(y_{cl})$	(*x*_*c*/*o*_)_0.1_
Feat. Imp.	0.20(±0.02)	0.16(±0.02)	0.20(±0.02)	0.20(±0.02)	0.25(±0.02)

## Discussion

It is well known that CoP platforms have been widely used by clinical researchers in order to further evaluate the postural control of investigated populations [11, 13]. The features selection has been based on previous works which reported that statokinesigrams medio-lateral and antero-posterior ranges, trajectories’ velocities and accelerations, mean distance from centre of the CoP trajectory [3, 16, 18], as well as more advanced local indices such as distances between peaks of sway density curve (ballistic intervals) [17, 20], might help to predict future falls from CoP trajectories.

In our population, all but one features did not show significant results through the conventional Wilcoxon test, in line with previous works [3, 16, 18], and their performances in ROC analysis was close to the performance of a random classifier (AUC between 0.49-0.54), questioning the features’ ability to successfully classify the two groups (Table 3). The reason for these results might be dual. Firstly, in such complex phenomena such as postural control, there might not be a simple heuristic which can completely characterize the aforementioned phenomenon. Fig 1 shows characteristic statokinesigrams of fallers and non fallers, illustrating the difficulties in distinguishing fallers by non-fallers. Differences between Fig 1a/1b and 1c/1d (faller/non-faller) cannot be easily exploited using only conventional single-feature analyses. The combined increase of expressive power resulting from the combination of multiple features and non linear methods seems to be more suitable to differentiate fallers from non fallers. Secondly, the results from conventional statistical tests should be treated carefully. Tests such as the Wilcoxon ranksum test do reply to the significance of the difference between the distributions of two groups. However, a statistical difference between two distributions does not necessarily imply an efficient solution to the classification problem, except for the easiest cases (e.g. distributions with minimal overlap), which is not the case for faller/non-faller classification. Therefore, additional information is needed in order to classify a new participant in faller or non-faller group (prediction).

The aforementioned results illustrate the fact that the combination of multiple features can partially indicate postural instability. These arguments are in line with the concerns of previous works [12, 14] about the ability of single features, despite of their usefulness (e.g. ML movement), to distinguish fairly accurately a faller by a non faller.

Previous works also involved multiple indices in their instability evaluation models [29, 30]. It was reported that the analysis of multi-dimensional characteristics using machine learning approaches might increase significantly the classification’s performance [31, 32]. These approaches are logically in line with the knowledge that every individuals’ physiological characteristic may affect the statokinesigram simultaneously, differently and relatively to other characteristics. More particularly, [29] already proposed a scoring approach based on multiple approaches. Their scoring approach, while promising, resulted in a lower AUC. It is the authors’ belief that this difference in performance can be explained by their algorithm which uses a simple linear combination of the features. Indeed, the nature of the interrelation of the features might be arbitrarily complex, and linear model can fail to encode those relations. The deeply non-linear nature of the Ranking Forest algorithm allows it to encode more general models (see e.g. [23]).

The above reasons justify our strategy to use a multi-dimensional characterization of statokinesigrams and the non-linear Ranking Forest algorithm in order to disentangle fallers from non-fallers sways. An online version of the resulting scoring algorithm, which can be applied to the existing dataset or to new statokinesigrams, can be found at http://taureau.pppcmla.ens-cachan.fr/.

### Limitations

A limitation in our work might be the fact that due to the current available dataset our approach cannot give further information about the physiological reasons of the risk of fall. Previous works questioned the ability of the simple statokinesigram measurement to predict falling as well as to give reasons of fall due to the high simplicity of the measurement and also due to lack of realistic conditions [33]. It has been mentioned that in individual’s real life, falls often occurs when multiple tasks or external stimulations exists simultaneously and fragile individuals cannot process them accurately [12]. Therefore, they proposed richer and more dynamic protocols involving multiple tasks [19, 33]. Increase of exercises’ difficulty such as stance in foam surface were also proposed [3]. However, the primary purposes of this work was to highlight that: (1) simple statokinesigrams may contain sufficient information for a first classification of elderly fallers/non-fallers and, (2) the latter information can be extracted using multi-dimensional statokinesigrams’ characterization and advanced data-mining strategies. We agree that richer protocols can not only lead to more accurate and in-depth faller/non-faller classification, but also to more fruitful statokinesigram traces of particular individual’s physiological impairments (visual, vestibular,somatosensor, nervous system). Recent works reported that force platforms and body accelerometers might be a powerful combination in evaluating the risk of falls and identifying the underlying physiological reasons [18, 34]. Further investigation should be done in methodologies which are able to fully exploit the richness of such protocols.

Another limitation might be the choice of the Wii Balance Board as a force platform. Despite the increasing popularity of the Wii Balance Board in posturography, (see e.g. [35]), many researchers have argued against the use of the Wii Balance Board for postural control assessment (see e.g. [36]). The main shortcomings of the Wii Balance Board are its lower accuracy, its inconsistent sample rate and its inferior signal to noise ratio, compared to gold-standard, which makes the Wii Balance Board a less reliable out-of-the-box device to measure postural control. Accordingly the Wii Balance Board was found to have a modest agreement with laboratory grade force platforms [37]. However, recent works such as [25] and [27] have shown that with proper preprocessing, the data collected from the Wii Balance Board was more accurate, and closer to laboratory grade force platforms. Additionally, it is the authors’ belief that the method developed in our work can be successfully applied to laboratory grade force-platform that are more accurate that the Wii Balance Board, since the resulting statokinesigrams will be of higher quality and thus will contain even more information.

## Conclusion

In this study, a new approach for classifying fallers/non-fallers in an community-dwelling elderly population (84 individuals) was proposed. This approach, based on the Ranking Forest algorithm, combines the advantages of having robust classification performances while using only two simple static sway measurements. In this model, the statokinesigrams are characterized in a multi-dimensional space (five features) and evaluated with a non-linear scoring algorithm which has been trained using a subset of 70% of the overall dataset (training set). Its performance was validated in the rest 30%. The results were compared with the performance of each feature separately showing that although each feature has almost a random performance in classifying fallers/non-fallers, the Ranking Forest score reached significantly higher performance. As a consequence, in our population, significant information about the prediction of risk of future fall can be extracted even through simple, one minute long protocols. It is the authors’ belief that this approach, after some appropriate modifications, can be applied also to other protocols. The results of the proposed method are promising and can also be of major usefulness in evaluation of postural instability severity in widespread-diseases such as Parkinson’s disease.
